# Proteomic Analysis of *Auricularia auricula-judae* Under Freezing Treatment Revealed Proteins and Pathways Associated With Melanin Reduction

**DOI:** 10.3389/fmicb.2020.610173

**Published:** 2021-01-15

**Authors:** Jiawen Li, Ziwei Li, Tong Zhao, Xiufeng Yan, Qiuying Pang

**Affiliations:** ^1^Key Laboratory of Saline-alkali Vegetation Ecology Restoration, Ministry of Education, College of Life Sciences, Northeast Forestry University, Harbin, China; ^2^College of Life and Environmental Science, Wenzhou University, Wenzhou, China

**Keywords:** *Auricularia auricula-judae*, freezing treatment, proteomics, mycelium, fruiting bodies

## Abstract

*Auricularia auricula-judae* is an edible nutrient-rich mushroom, which is a traditional medicinal resource in China. It is known that environment stimuli will affect the production of melanin by *A. auricula-judae*, but the mechanism of the effects of freezing treatment on melanin accumulation remains unknown. In the present study, the synthesis of melanin in *A. auricula-judae* was analyzed by physiological assays and a proteomics approach. Our findings showed that a longer freezing treatment causes a lighter color of *A. auricula-judae* fruiting bodies. The proteomic analysis showed that proteins involved in glycolysis/gluconeogenesis, tyrosine metabolism, ribosome, and arginine biosynthesis might contribute to the color differences in the *A. auricula-judae* after freezing treatment. This work will be expected to provide valuable information on the physiological and molecular mechanisms of freezing treatment on the color quality of *A. auricula-judae*.

## Introduction

*Auricularia auricula-judae* is an edible nutrient-rich mushroom, which is an important food resource in Asia due to it being rich in proteins, microelements, vitamins, and carbohydrates as well as its low fat content (Luo et al., 2009). *A. auricula-judae* is also known for its medicinal properties, such as its antitumor, detoxification, anticoagulant, hypoglycemic, and cholesterol-lowering effects (Nguyen et al., 2012). Wild *A. auricula-judae* is geographically distributed in Asia, Europe, North America, and other places throughout temperate and subtropical regions (Akpaja et al., 2005). *A. auricula-judae* has been cultivated on a large scale in northern China, with a yield that ranked fourth in the world (Zhang et al., 2007). With the improvement of the nutritional and medicinal values of *A. auricula-judae*, its harvesting method has gradually evolved from wild collection to artificial cultivation.

*Auricularia auricula-judae* is widely cultivated in China, and northern China was the major cultivation area (Li et al., 2019). In 2012, its production in Heilongjiang was more than 3,000,000 tons, with an output value of more than 20 billion yuan (Liu and Hu, 2018). *A. auricula-judae* is mainly cultivated by two different methods, wood cultivation and bagging cultivation (Lv, 2008). Compared with wood cultivation, bagging cultivation (Supplementary Figure 1) is more widely used because of the lower expenses, including wood resources and economic investment, as well as the avoidance of pesticides and viruses (Lv, 2008). The freezing pretreatment of the mycelium is a common method and key step of bagging cultivation in northern China, and when producing cultivation species of *A. auricula-judae* in the winter, the cultivation species full of mycelium are put outdoors for quick-freezing, followed by waiting for ear emergence in the spring. Freezing treatment can significantly improve the yield and quality of *A. auricula-judae* (Gao, 2012), but the fruit color has been greatly affected, which is the most important indicator to estimate the food quality of *A. auricula-judae*. The color of *A. auricula-judae* fruiting bodies turned to yellow after the mycelium was frozen. However, we are still far from understanding the physiological and molecular bases of this phenomenon.

*Auricularia auricula-judae* melanin belongs to the 3,4-dihydroxyphenylalanine (DOPA) melanin, and it was initially determined to be pheomelanin by Sephadex G-100 column chromatography, which is a complex polyphenolic compound (Zou et al., 2013). DOPA-based melanin is a natural pigment widely found in living organisms, and the hair of humans (*Homo sapiens*), bones and feathers of *Gallus*, seed coat of *Armeniaca sibirica*, and black truffles (*Tuber melanosporum*) contain a large amount of DOPA-like melanin (Butler and Day, 1998; Yao et al., 2007). The products in the biosynthesis process of DOPA melanin are divided into eumelanin (brown or black) and pheomelanin (brown, red, or yellow) (Zou et al., 2013). The color of melanin to eumelanin or pheomelanin during synthesis depends on the tyrosinase activity and substrate concentration of cysteine (Simon et al., 2009). When cysteine is present in the cell, dopaquinone (DQ) will immediately bind to cysteine to form cysteamine DOPA, and when most of the cysteamine DOPA in the cell is consumed, DOPA will undergo its own cyclization reaction to form a cyclized DOPA, eventually synthesizing pheomelanin (Ito and Wakamatsu, 2003). Studies have shown that tyrosinase activity is significantly reduced and even inactivated, which will directly affect the production of melanin by *A. auricula-judae* under strong-light, high-temperature, or cold conditions (Geib et al., 2016).

Until now, the quality changes of *A. auricula-judae* caused by environmental factors have not been studied using a comprehensive approach, such as proteomics analysis. Thus, this study will detect the protein expression profile to explore the physiological mechanism of the effects of freezing treatment on the color quality of *A. auricula-judae*. We aim to study the differences in protein abundances due to freezing treatment in *A. auricula-judae* and identify the roles of the identified differentially expressed proteins in the melanin biosynthesis process. Our findings are expected to provide a valuable picture of the physiological and molecular mechanisms of freezing treatment on the color quality of *A. auricula-judae* and provide a resource for the identification of key proteins contributing to the improvement in the concentration of melanin in *A. auricula-judae*.

## Materials and Methods

### Sample Treatment and Collection

*Auricularia auricula-judae* (HW15) fruiting bodies are single monolithic; rootless; moderate in size; shaped like a shell or a bowl with round, thick, and soft edges; gray on the back of the dry ear; black on the abdomen; and shiny, with a big color difference between the front and the back and with an inconspicuous ear vein, and they were obtained from the experimental forestry farm of the Heilongjiang Academy of Forestry (Dai et al., 2014). They are cultured on a substitute medium in mycelium bags containing 78% saw dust, 20% bran, and 2% gypsum powder. The relative water content of the substitute medium was 65%. All mycelium bags were stored at 25°C in the mycelium growing season until the surface of the mycelium bags became white, which meant that the mycelium was mature. Then, they were stored at 4°C, and some mycelium bags were transferred outdoors as freezing treatment (Supplementary Figure 2). All fruiting bodies were quickly frozen in liquid nitrogen, ground to a fine powder using a mortar and pestle, and stored at −80°C before protein extraction.

### Color Measurement

The International Commission on Illumination (CIE) color system was applied to study the color changes of fruiting bodies during different levels of the freezing treatment. The CIE recommended the CIE L^∗^a^∗^b^∗^ color system in 1976 as a standard to describe an objective color (Takeshi et al., 2016). The color of fruiting bodies was measured by Color Testers (CR-10, Konica Minolta). The L^∗^ value indicates lightness (0–100 represent dark color–light color). The a^∗^ coordinate indicates redness and greenness in the positive (+a^∗^) and negative (−a^∗^) directions, respectively. The b^∗^ coordinate indicates yellowness and greenness for the positive (+b^∗^) and negative (−b^∗^) directions, respectively. When b^∗^ is positive, it is only related to the yellow color. The larger the b^∗^ value is, the yellower the color (Zielinska and Michalska, 2016).

### Enzyme Activity Assay

Mycelium and fruiting bodies were ground to a fine powder in liquid nitrogen, fully mixed in extraction buffer, and centrifuged at 4°C at 15,000 × *g* for 20 min. The supernatant was then extracted for enzyme activity detection. Tyrosinase, laccase, superoxide dismutase (SOD), and peroxidase (POD) activities were examined by an ultraviolet (UV) spectrophotometer (Wang et al., 2013; Zhang et al., 2013).

### Mineral Element Analysis

Inductively coupled plasma–optical emission spectroscopy (ICP-OES, Optima 8300, Perkin Elmer) was used to detect the mineral elements in the mycelia and fruiting bodies of *A. auricula-judae*. The standard sample was purchased from the National Center of Analysis and Testing for Nonferrous Metals and Electronic Materials (China). The solution for element detection was prepared by ash nitration method. Fine powder was obtained by grinding the mycelia and fruiting bodies after drying, carbide samples were thereafter transferred to a muffle furnace (SX-4-10, China), ash treated at 625°C, and then dissolved with 2% HNO_3_. ICP-OES operating conditions were as follows: plasma gas flow rate of 14 L⋅min^–1^, auxiliary gas flow rate of 0.2 L⋅min^–1^, atomizer gas flow rate of 0.55 L⋅min^–1^, RF power of 1200 W, and sample flow rate of 1.5 mL⋅min^–1^.

### Protein Extraction

The total protein was extracted from 10 g of fruiting bodies and 500 mg of mycelium, according to the phenol-based protocol referring to Li et al. (2018). The fine powder was homogenized in extraction buffer (0.9 M sucrose, 0.1 M Tris-HCl, 10 mM EDTA, 1% PVPP, 0.4% β-mercaptoethanol, and Tris-phenol) for 0.5–2 h until the mixture turned into liquid, followed by centrifugation at 20,000 × *g* for 20 min at 4°C and the collection of the phenol phase. The phenol phase was precipitated with five volumes of cold 0.1 M ammonium acetate in methanol for 20 min at -20°C, and then, the precipitate was collected by centrifugation at 20,000 × *g* for 20 min at 4°C. After washing with 0.1 M ammonium cold acetate and 80% acetone successively, pure protein powder was obtained. The protein powder was dissolved in lysis buffer (7 M urea, 2 M thiourea, 4% 3-[(3-cholamidopropyl) dimethylammonio]propanesulfonate (CHAPS), 40 mM dithiothreitol (DTT), 2% pharmalyte, and 4% protease inhibitor). The protein concentration was determined using the 2-D Quant Kit (GE Healthcare, Little Chalfont, United Kingdom) with BSA as the standard. Samples were frozen in liquid nitrogen and kept at -80°C for further use.

### Two-Dimensional Gel Electrophoresis (2-DE) and Image Analysis

Two-dimensional electrophoresis (2-DE) of protein samples was performed as described previously (Li et al., 2018). A protein sample of 1300 μg was loaded by rehydration to immobilize DryStrip gels (pH = 3–10 linear, 24 cm) (GE Healthcare, Waukesha, WI, United States) individually. After automated detection and matching, manual editing was carried out to correct the mismatched and unmatched spots. Spots were considered reproducible when they were well resolved in the three biological replicates. For each matched spot, a measurement was carried out for each biological replicate, and normalized volumes were computed using the total spot volume normalization procedure of the software. The normalized volume of each spot was assumed to represent its expression abundance. A criterion of *p* < 0.05 and an abundance ratio of at least 1.5 were used to define significant differences when analyzing parallel spots between different groups with two-way ANOVA.

### Protein Identification by Mass Spectrometry

Selected spots were excised from 2-DE gels, washed with sterile deionized water, and digested with trypsin as described previously (Chen et al., 2011). The obtained dry protein was redissolved in 0.1% trichloroacetic acid and identified by mass spectrometry. MS/MS spectra were acquired using an ABI 5800 MALDI-TOF/TOF Plus mass spectrometer (Applied Biosystems, Foster City, CA, United States). Data were acquired in a positive MS reflector using a CalMix5 A standard to calibrate the instrument (ABI5800 Calibration Mixture). Primary and secondary mass spectrometry data were integrated, and the results were analyzed and identified using GPS 3.6 (Applied Biosystems) and Mascot 2.3 (Matrix Science). Identification of proteins was performed using the following search parameters: NCBI-*Auricularia*, NCBI-fungi database, trypsin as the digestion enzyme and allowable maximum leakage cutting locus of 1, fixed modification of carbamidomethyl (C), partial modifications of acetyl (protein N-term), deamidated (NQ), dioxidation (W), and oxidation (M), the MS tolerance was 100 ppm, the MS/MS tolerance was 0.3 Da, and a protein score CI% of more than 95% indicates successful identification.

### Functional Categorization of Proteins

The Kyoto Encyclopedia of Genes and Genomes (KEGG) (last updated on 1 November 2020) (Kanehisa et al., 2008) is a database resource for understanding high-level functions and utilities of a biological system, such as the cell, the organism, and the ecosystem, from molecular-level information, especially large-scale molecular datasets generated by genome sequencing and other high-throughput experimental technologies^1^. We used KOBAS software to test the statistical enrichment of differentially expressed proteins in KEGG pathways.

### UV–Visible/Fourier Transform Infrared (FTIR) Spectra of *A. auricula-judae* Melanin Analysis

The powder of freeze-dried melanin is mixed with KBr (mass ratio 1:200) and pressed into tablets, and the infrared (IR) spectrum (500–4000 cm^–1^) is recorded by a Fourier transform infrared (FTIR) spectrometer (Zou et al., 2013).

### Electron Paramagnetic Resonance (EPR) Analysis of *A. auricula-judae* Melanin Analysis

The melanin of *A. auricula-judae* was extracted with NaOH and HCL and purified by using strong acid hydrolysis and organic solvent (Li, 2011). The purified melanin of *A. auricula-judae* was identified by EPR.

### qRT-PCR Analysis

To validate the protein data, nine genes were chosen for RT-PCR analysis. The total RNA was isolated with TRIzol reagent (Invitrogen) according to the manufacturer’s instructions and then treated with recombinant DNase I (RNase-free, TaKaRa, Tokyo, Japan). Ten micrograms of RNA was used to synthesize the cDNA with the PrimeScript RT Reagent Kit (TaKaRa, Tokyo, Japan), and the cDNAs were used for RT-PCR analysis with specific primers (Supplementary Table 1). Quantitative RT-PCR was performed using the Applied Biosystems 7500 Real-Time PCR System using Power SYBR Green chemistry (TaKaRa, Tokyo, Japan). 18S was quantified as an internal control, and the 2^–ΔΔCt^ method was used to analyze differential expression (Livak and Schmittgen, 2001). The mean of three biological replicates, for which three technical replicates were averaged, was presented. Genes were considered to be differentially expressed if the fold change was ≥1.5 or ≤0.67, and the *p*-value was tested by ANOVA with the *post hoc* Tukey test (*p* < 0.05).

### Statistical Analysis

All statistical calculations were performed by the SPSS 17.0 software package for Windows (SPSS Inc., Chicago, IL, United States). All claims of statistical significance (*p* < 0.05) were assessed by Student’s *t*-test and two-way ANOVA (*p* < 0.05) with Tukey HSD *post hoc* test.

## Results

### Color Analysis

**FIGURE 1:**
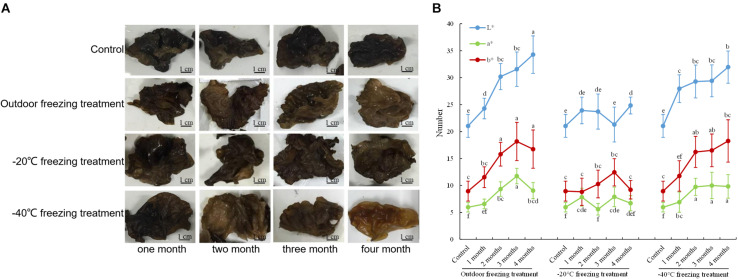
Color changes of *A. auricula-judae*. **(A)** The color phenotype of fruiting bodies under outdoor, −20°C, and −40°C freezing treatments for 1–4 months. **(B)** The CIE L^∗^-value (lightness), CIE a^∗^-value (redness), and CIE b^∗^-value (yellowness) of the fruiting bodies of *A. auricula-judae* were determined under outdoor, −20°C, and −40°C freezing treatments. Different letters indicate statistically significant differences between the means (*p* < 0.05) for L^∗^, a^∗^, and b^∗^ calculated by one-way ANOVA.

Under the treatments of outdoor freezing and −40°C freezing, the color of the fruiting bodies was lighter with longer processing times. The color of fruiting bodies is not significantly different under different times of -20°C freezing treatment (Figure 1A). The CIE test showed a greater L^∗^ value of the fruiting bodies under different times of outdoor and -40°C freezing treatments. After the outdoor and -40°C freezing treatments, the b^∗^ value of the fruiting bodies was increased after 2 months of freezing treatment and had no change after 3 or 4 months of treatment compared to 2 months. Under −20°C freezing treatment, the L^∗^ and b^∗^ values of fruiting bodies did not continuously increase with the change of treatment time. The a^∗^ value of the fruiting bodies showed no significant alteration under three freezing treatments (Figure 1B). Combining the color observation and CIE test, we found that the color of fruiting bodies was significantly changed under 2 months of outdoor freezing treatment, which was quite brown compared to the control group, and the *A. auricula-judae* with outdoor freezing treatment for 2 months was used for the follow-up analysis. According to the phenotypic observation of *A. auricula-judae*, the biomass of the *A. auricula-judae* significantly increased under freezing treatment, which is consistent with previous research (Gao, 2012).

### Enzyme Activity Analysis

**FIGURE 2:**
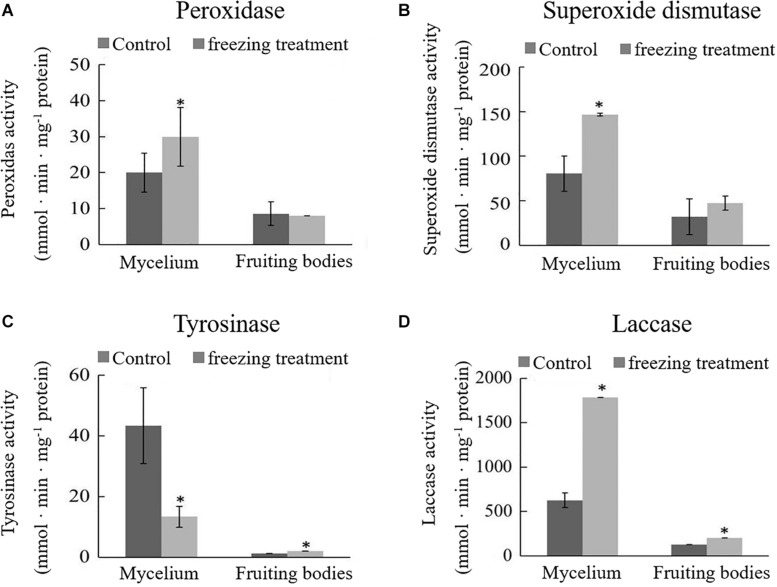
Effects of outdoor freezing treatment on the enzyme activities of *A. auricula-judae* mycelium and fruiting bodies. The significant differences in the enzyme activities of **(A)** peroxidase, **(B)** superoxide dismutase, **(C)** tyrosinase, and **(D)** laccase between control and freezing-treated groups are indicated by an asterisk (*p* < 0.05) under outdoor freezing treatment. Values represent the means ± SDs (*n* ≥ 3). Data were analyzed using Student’s *t*-test.

During the freezing treatment, the changes in ambient temperature may cause oxidative stress, which affects the synthesis of DOPA melanin (Jarrar et al., 2017). To test if the color change of *A. auricula-judae* upon freezing treatment correlated with oxidative stress, we detected the activities of POD and SOD, the main antioxidative enzymes that would be induced by oxidative stress. The activities of POD and SOD in the mycelium were significantly increased after outdoor freezing treatment. In contrast, there is no significant difference comparing the control and treated groups in the fruiting bodies (Figures 2A,B). This indicated that the freezing treatment caused oxidative stress to the mycelium, which may affect the synthesis of melanin in the mycelium.

Tyrosinase and laccase are known to play a key role in melanin synthesis (Sapmak et al., 2015). We therefore analyzed whether freezing treatment could impact the tyrosinase and laccase enzyme activities. The activity of tyrosinase was inhibited, and laccase activity was enhanced in the mycelium after freezing treatment (Figures 2C,D), which indicates that freezing treatment inhibits the melanin synthetic process. The activities of tyrosinase and laccase in the fruiting bodies were significantly higher than those in the control group and far lower than those in the mycelium; therefore, we infer that the melanin synthesis begins in the mycelium stage. L-DOPA was synthesized from tyrosine, which was catalyzed by tyrosinase and then further became DQ in the mycelium stage. DQ may react with cysteamine or glutathione at the fruiting bodies stage for further oxidative polymerization to produce pheomelanin.

### Mineral Element Analysis

**FIGURE 3:**
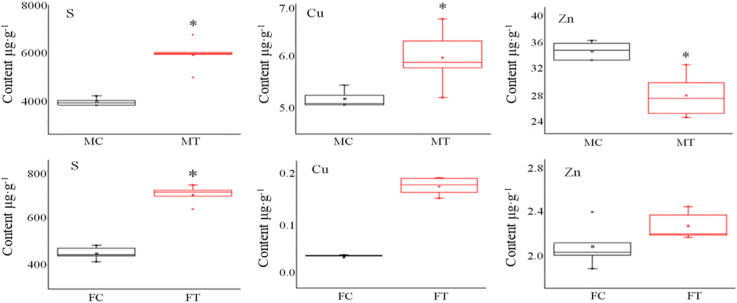
The influence of freezing treatment on the content of mineral elements in *A. auricula-judae* mycelium and fruiting bodies. MC represents the mycelium control group. MT represents the mycelium treatment group. FC represents the fruiting body control group. FT represents the fruiting body treatment group. Black indicates control. Red indicates freezing treatment. An asterisk (^∗^) represents a significant level of difference (*p* ≤ 0.05).

Pheomelanin is a sulfur-containing macromolecule, a melanin synthesized by tyrosine, dopamine, dopamine, tyramine, cysteine, and glutathione (Prota, 1980). Some metal ions can activate or inhibit protease activities, such as high concentrations of Cu and Zn that can inhibit the activity of tyrosinase and laccase (Chen and Song, 2006). Thus, we detected S, Cu, and Zn elements by ICP-OES analysis. As shown in Figure 3, it was found that the contents of S and Cu elements in the mycelium were increased, but Zn was decreased after freezing treatment. Different situations happened in the fruiting bodies; the accumulation of S, Cu, and Zn was all significantly induced by freezing treatment (Figure 3), suggesting that the inhibition of melanin synthesis caused by freezing condition may be correlated with these elements.

### Protein Identification

**FIGURE 4:**
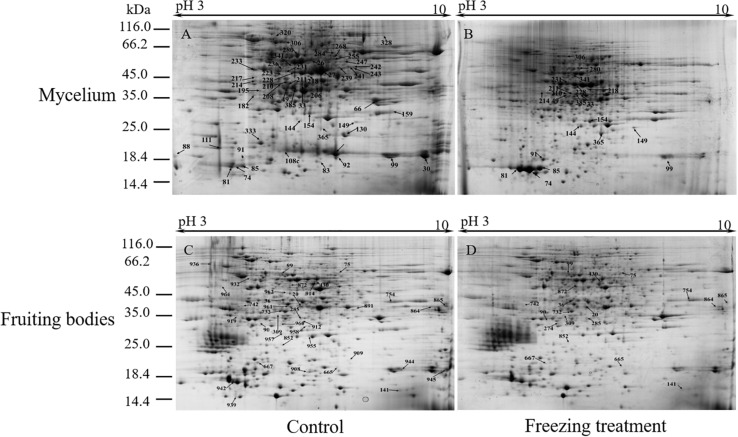
Representative 2D images of the *A. auricula-judae* mycelium and fruiting body proteome in response to outside freezing treatment. Spot distribution of the proteins isolated from mycelium under control **(A)** and freezing treatment **(B)**, fruiting bodies under control **(C)** and freezing treatment **(D)** conditions. Identified differentially expressed protein spots are indicated by arrows and number labeled. IEF was performed with the total protein extract of *A. auricula-judae* using a 24-cm pH 3–10 IPG strips, followed by SDS-PAGE with Coomassie Blue R250 protein gel stain. The numbers refer to the proteins that were successfully identified and are shown in Tables 1, 2. The molecular masses of the Precision Plus Protein Kaleidoscope standards (Bio-Rad) are indicated on the left.

**FIGURE 5:**
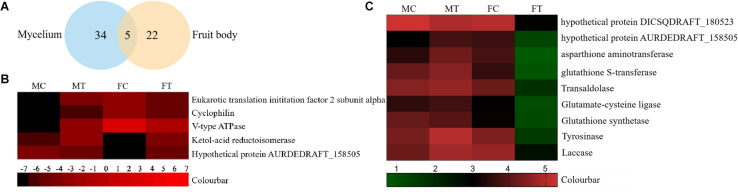
The differentially expressed proteins in *A. auricula-judae* mycelium and fruiting bodies under freezing treatment. **(A)** A Venn diagram shows the overlap of differentially expressed proteins between the mycelium and fruiting bodies in *A. auricula-judae*. **(B)** A heat map represents the relative expression levels of proteins under freezing treatments. Blocks with colors indicate downregulated (black) or upregulated (red) expression. **(C)** Quantitative RT-PCR analysis of the gene expression of correlated encoding proteins in *A. auricula-judae* mycelium and fruiting bodies. Each data point represents the mean ± SE (*n* = 3). MC represents the mycelium control group. MT represents the mycelium treatment group. FC represents the fruiting bodies control group. FT represents the fruiting bodies treatment group.

To analyze the underlying molecular network contributing to the regulation of the color change of *A. auricula-judae* under freezing treatment, a comparative proteomics analysis was performed, and the protein expression profile in *A. auricula-judae* was uncovered. We identified more than 1500 protein spots in the CBB staining 2D gels by the detection of ImageMaster 2D Platinum software. The isoelectric point of the protein is in the pH range of 3–10, and the molecular weight is between 14.4 and 1116.0 kDa (Figure 4). Based on the fold change of more than 1.5 with a *p*-value < 0.05 of variation in protein abundance, we detected a total of 51 and 36 differentially expressed protein spots in the mycelium and fruiting bodies after freezing treatment (Tables 1, 2). As shown, all proteins identified were supported by the MS/MS sequenced peptides. A total of 73 proteins were successfully identified according to peptide matching results using MASCOT (Supplementary Table 2). Among the 73 differentially expressed proteins, 12 proteins were upregulated and 27 proteins downregulated in the mycelium, while eight proteins were upregulated and 19 proteins downregulated in the fruiting bodies (Figure 5A); meanwhile, seven proteins in the mycelium and fruiting bodies failed to be identified. Among these distinct proteins, it was found that the expression of tyrosinase and laccase in the mycelium and fruiting bodies had no change after freezing treatment. This means that the regulation of the activities of these two melanin synthases by freezing treatment may not result from the change of protein abundance. There were five proteins that exhibited changed expression in both the mycelium and fruiting bodies. The expression levels of ketol-acid reductoisomerase in the mycelium and fruiting bodies were similar, and the protein expression levels were upregulated after freezing treatment. The other three proteins, eukaryotic translation initiation factor 2 subunit alpha (EIF2S1), cyclophilin, and V-ATPase, were upregulated in the mycelium after freezing treatment but downregulated in the fruiting bodies. In contrast, the expression level of hypothetical protein AURDEDRAFT_158505 was downregulated in the mycelium but upregulated in the fruiting bodies after freezing treatment (Figure 5B). This means that the physiological and mechanistic effects of freezing treatment on the mycelium and fruiting bodies of *A. auricula-judae* may be distinct.

**TABLE 1:** Protein identities and their relative changes under freezing treatment in *A. auricula-judae* mycelium.

**Spot No.^a^**	**NCBI accession No. (GI)^b^**	**Protein name**	**Exp pI/kDa^c^**	**Thero pI/kDa^d^**	**Score^e^**	**SC^f^^(^%)**	**PN^g^**	**Mycelium fold change**
**Amino acid metabolism**								
26	gi| 393240892	Iron-containing alcohol dehydrogenase 1, partial	6.60/44.38	7.75/53.19	107	2	1	A
218	gi| 393242110	Ketol-acid reductoisomerase	6.89/35.21	8.83/43.89	58	2	1	3.33
231	gi| 393246451	3-Isopropylmalate dehydrogenase	6.18/36.54	5.41/40.22	202	8	2	6.71
236	gi| 393246451	3-Isopropylmalate dehydrogenase	5.82/40.90	5.41/40.22	205	8	2	A
241	gi| 393245569	Aspartate aminotransferase	7.52/40.90	8.23/46.37	90	5	2	A
242	gi| 393246788	NAD(P)-binding protein	7.71/40.43	8.31/36.96	221	8	2	A
268	gi| 393237973	Acetylglutamate kinase ARG6	7.23/45.38	8.40/94.44	168	3	2	A
328	gi| 393245388	Trehalose phosphorylase	8.51/66.2	6.39/82.73	310	7	5	A
**Carbohydrate metabolism**								
27	gi| 393241500	Citrate synthase	6.70/40.43	7.77/51.61	100	6	2	A
33	gi| 393242616	Transaldolase	6.31/34.89	6.62/35.77	70	3	1	0.20
210	gi| 393236505	Fructose-1,6-bisphosphatase	6.32/34.68	5.67/37.88	103	4	1	0.39
214	gi| 393246070	Thiamin diphosphate-binding protein	5.19/34.88	4.98/36.16	319	16	4	0.27
284	gi| 393234096	Hypothetical protein AURDEDRAFT_90114	7.21/50.87	6.24/56.62	146	8	3	A
**Energy metabolism**								
74	gi| 393245928	V-type ATPase	4.71/14.48	5.75/68.67	40	2	1	14.57
81	gi| 393245928	V-type ATPase	4.55/14.49	5.75/68.67	43	2	1	6.13
91	gi| 393245928	V-type ATPase	6.32/34.45	5.75/68.67	42	2	1	2.76
385	gi| 393246905	Pyrophosphatase-domain-containing protein	6.83/32.10	5.70/32.97	58	4	1	4.76
**Genetic information processing**								
159	gi| 393247912	Ribosomal protein L13e	8.63/25.38	11.45/23.64	154	16	3	A
195	gi| 393229618	Ubiquitin, partial	5.38/32.34	6.81/8.94	285	36	3	A
211	gi| 393246789	Bet v1-like protein	6.39/34.23	5.30/34.44	60	6	2	0.36
217	gi| 393234431	Eukaryotic translation initiation factor 2 subunit alpha	5.17/35.23	5.00/35.87	99	4	1	A
247	gi| 393232408	26S proteasome subunit P45	7.42/43.89	6.00/41.63	75	4	1	A
280	gi| 393234431	Eukaryotic translation initiation factor 2 subunit alpha	6.25/46.47	5.00/35.87	41	4	1	0-
306	gi| 393247433	Hypothetical protein AURDEDRAFT_178815	6.32/63.88	5.58/70.71	73	1	1	0.33
341	gi| 393242042	Galactose mutarotase-like protein	6.21/41.89	5.64/46.03	83	4	2	11.12
**Metabolism of cofactors and vitamins**								
144	gi| 393245268	Hypothetical protein AURDEDRAFT_158505	6.351/24.37	6.99/22.90	73	5	1	4.79
**Nucleotide metabolism**								
206	gi| 393226636	NAD(P)-binding protein	6.891/33.45	6.37/36.60	68	3	1	A
**Transport and catabolism**								
149	gi| 393228574	Manganese superoxide dismutase	7.56/24.31	6.65/22.44	216	15	3	A
365	gi| 393228574	Manganese superoxide dismutase	6.71/24.89	6.65/22.44	208	15	3	A
**Glycan biosynthesis and metabolism**								
83	gi| 9586355	Glycoside hydrolase family 38 protein	6.87/13.56	5.83/119.98	83	0	1	A
**Lipid metabolism**								
49	gi| 393246546	Prolyl aminopeptidase serine peptidase	6.42/32.87	5.60/35.70	70	4	1	0.55
320	gi| 393234249	Hypothetical protein AURDEDRAFT_153044	5.61/66.54	6.53/79.80	86	3	2	A
**Metabolism of other amino acids**								
154	gi| 393240170	Glutathione *S*-transferase	6.56/24.88	6.43/23.47	78	4	1	2.26
**Metabolism of terpenoids and polyketides**								
182	gi| 393247824	Isopentenyl diphosphate isomerase	9.211/31.87	5.22/28.86	86	10	2	A
233	gi| 393247674	GTP binding protein	5.21/20.68	5.06/41.44	420	16	5	A
**Unknown**								
85	gi| 393244469	YjgF-like protein	5.51/14.49	5.61/14.18	34	7	1	0.44
88	gi| 393238524	Hypothetical protein AURDEDRAFT_63592	3.00/15.45	5.27/13.16	36	1	2	A
99	gi| 393230623	Cyclophilin	8.52/15.45	8.78/17.49	334	27	3	2.76
208	gi| 393235619	Hypothetical protein AURDEDRAFT_126065	5.83/34.15	10.97/45.84	37	2	1	A
223	gi| 393243746	Protein prenyltransferase	5.81/36.78	5.58/39.04	163	10	3	A
228	gi| 393243670	Putative cyanide hydratase	6.21/36.58	5.96/40.75	172	10	3	4.58
239	gi| 393227903	Carbohydrate-binding module family 12 protein	7.31/40.88	6.18/36.27	64	8	3	A
243	gi| 393245114	Hypothetical protein AURDEDRAFT_111257	7.71/40.51	7.93/11.14	43	15	1	A
255	gi| 393241515	Proliferation-associated protein 1	7.23/43.25	7.61/42.43	147	7	3	A

*Two treatments including CK (control check) and OS (2 months outside freezing treatment) were performed; protein spots showing significant differences (*n* = 3, 1.5-fold, *p* ≤ 0.05) were cut out and identified by LC-MS. ^*a*^Assigned spot number as indicated in Figures 4A,B. ^*b*^Database accession numbers according to NCBI database. ^*c*^Theoretical and ^*d*^experimental mass (kDa) and pI of identified proteins. Experimental values were calculated using ImageMaster 2D Platinum software. Theoretical values were retrieved from the protein database. ^*e*^Statistical probability of true positive identification of the predicted protein calculated by MASCOT. ^*f*^Sequence coverage. ^*g*^Number of peptides sequenced. ^*h*^Mean of relative protein abundance and standard error. “A” represents the protein spots deleted after treatment compared with the control group.*

**TABLE 2:** Protein identities and their relative changes under freezing treatment in *A. auricula-judae* fruiting bodies.

**Spot no.^a^**	**NCBI accession no. (GI)^b^**	**Protein name**	**Exp pI/kDa^c^**	**Thero pI/kDa^d^**	**Score^e^**	**SC^f^ (%)**	**PN^g^**	**Fruit body fold change**
**Genetic information processing**								
36	gi| 393243821	Serine/threonine-protein phosphatase PP1	5.81/37.231	5.78/38.48	107	11	3	5.88
99	gi| 393234431	Eukaryotic translation initiation factor 2 subunit alpha	5.73/47.11	5.00/35.87	41	4	1	2.58
667	gi| 393245529	L30e-like protein	5.28/21.33	5.38/16.36	175	21	3	0.43
732	gi| 393245027	Guanine nucleotide binding protein beta subunit	5.67/35.112	5.80/35.14	281	11	3	0.38
742	gi| 393241577	Serine/threonine-specific protein phosphatase Sit4	4.79/18.321	4.96/35.63	186	13	3	0.33
865	gi| 393232568	40S ribosomal protein S3	8.94/34.47	9.20/29.64	431	23	6	0.42
872	gi| 393235988	Hypothetical protein AURDEDRAFT_167263	5.9/45.21	5.96/43.11	73	3	1	0.32
909	gi| 393240875	60S ribosomal protein L35	7.2/24.14	10.79/14.80	41	10	1	A
945	gi| 393227063	Ribosomal protein L14b/L23e	9.47/20.11	10.17/15.15	137	14	2	A
**Transport and catabolism**								
90	gi| 393241397	Ras-like protein	5.34/33.32	5.09/24.68	182	12	2	2.11
**Carbohydrate metabolism**								
285	gi| 393240494	Phosphomannomutase	6.25/33.13	5.73/29.56	131	8	2	2.20
430	gi| 393246246	GroES-like protein	6.41/46.33	6.96/45.11	173	13	4	3.21
754	gi| 346995726	Glyceraldehyde 3-phosphate dehydrogenase	8.15/39.87	8.18/36.53	336	12	3	0.14
**Amino acid metabolism**								
309	gi| 18840885	Hypothetical protein DICSQDRAFT_180523	5.74/34.89	6.78/30.13	70	5	1	2.93
914	gi| 393246246	GroES-like protein	6.41/46.42	5.97/42.92	64	3	1	A
962	gi| 393242110	Ketol-acid reductoisomerase	5.89/43.14	8.83/43.89	146	5	2	A
**Energy metabolism**								
942	gi| 393245928	V-type ATPase	4.74/18.01	5.755/68.67	41	2	1	A
**Metabolism of cofactors and vitamins**								
955	gi| 393245268	Hypothetical protein AURDEDRAFT_158505	6.38/27.09	6.99/22.90	52	5	1	A
957	gi| 393245268	Hypothetical protein AURDEDRAFT_158505	5.89/27.51	6.99/22.90	52	11	3	A
**Nucleotide metabolism**								
958	gi| 393230751	Cleavage and polyadenylation-specific factor 5	6.26/36.89	6.23/23.90	38	7	1	A
**Signal transduction**								
961	gi| 393234194	Casein kinase I homolog 1	5.81/36.73	5.63/35.47	64	2	1	A
**Unknown**								
75	gi| 393242075	Peptidase C14	6.92/63.01	5.58/34.27	85	4	1	2.04
141	gi| 393243143	Chaperonin Cpn10	8.29/16.43	8.01/11.32	51	9	1	6.04
665	gi| 393230623	Cyclophilin	6.93/21.91	8.78/17.49	120	11	1	0.40
852	gi| 393246485	E set domain-containing protein	5.76/26.34	5.28/21.65	78	8	1	0.47
864	gi| 393243068	Voltage-dependent ion-selective channel	8.91/26.77	9.05/31.92	228	9	2	0.26
919	gi| 393246271	Hypothetical protein AURDEDRAFT_110532	4.92/34.89	4.89/21.82	142	9	1	A
944	gi| 393230623	Nucleoside diphosphate kinase	8.35/20.89	7.85/16.69	129	14	2	A
960	gi| 393230539	Hypothetical protein AURDEDRAFT_92020	5.87/32.22	6.28/73.05	120	5	2	A

*Two treatments including CK (control check) and OS (2 months outside freezing treatment) were performed; protein spots showing significant differences (*n* = 3, 1.5-fold, *p* ≤ 0.05) were cut out and identified by LC-MS. ^*a*^Assigned spot number as indicated in Figures 4C,D. ^*b*^Database accession numbers according to NCBI database. ^*c*^Theoretical and ^*d*^experimental mass (kDa) and pI of identified proteins. Experimental values were calculated using ImageMaster 2D Platinum software. Theoretical values were retrieved from the protein database. ^*e*^Statistical probability of true positive identification of the predicted protein calculated by MASCOT. ^*f*^Sequence coverage. ^*g*^Number of peptides sequenced. ^*h*^Mean of relative protein abundance and standard error. “A” represents the protein spots deleted after treatment compared with the control group.*

### Gene Expression

As proteomics studies showed, the protein expression of tyrosinase and laccase was not affected by freezing treatment in both the mycelium and fruiting bodies. This means that the regulation of the activity of these two melanin synthases did not depend on the protein abundance. To check if the differentially expressed proteins are correlated with their gene expression at the transcription level, nine genes were selected to perform the qRT-PCR analysis (Figure 5C). The expression of four transcripts in the fruiting bodies was consistent with the abundances of the corresponding proteins, including hypothetical protein AURDEDRAFT_158505, aspartate aminotransferase, glutathione S-transferase, and transaldolase. The transcription of glutathione S-transferase in the mycelium was consistent with the abundance of the corresponding protein. This indicated that the accumulation of these proteins is due to the upregulated mRNA expression. However, the transcript expression for the hypothetical protein AURDEDRAFT_158505, aspartate aminotransferase, and glutathione S-transferase in the mycelium and hypothetical protein DICSQDRAFT_180523 in the fruiting bodies showed contrary results to their protein abundances. The expression of these four genes was downregulated in the fruiting bodies and upregulated in the mycelium, except for glutathione synthetase (Figure 5C), and the expression changes in these proteins could be caused by glycosylation, protein phosphorylation, protein degradation, and ubiquitination under freezing treatment.

### Functional Annotation of the *A. auricula-judae* Proteome

**FIGURE 6:**
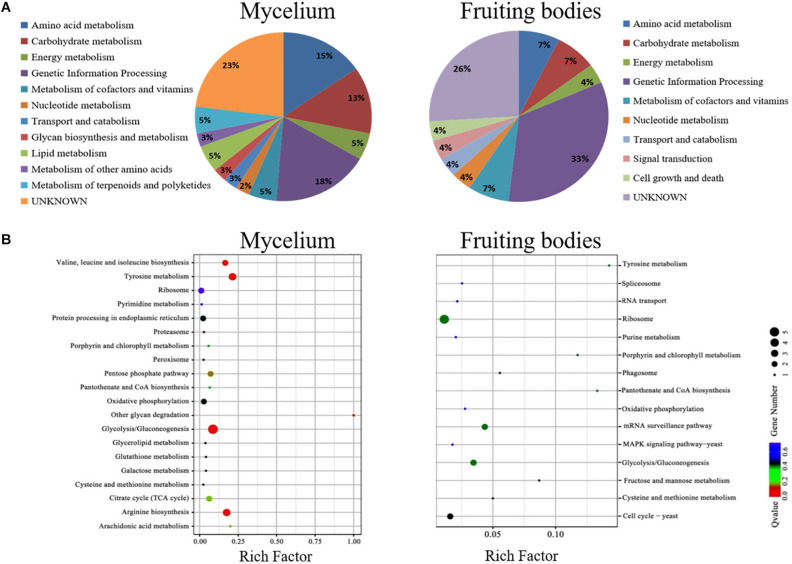
The COG and KEGG pathway enrichment of differentially expressed proteins. **(A)** The distribution of the functional categories of proteins expressed in the mycelium and fruiting bodies under freezing treatment. The pie chart displays the proportion of identified proteins assigned to different functional categories according to the COG functional classification. **(B)** KEGG pathway enrichment of differently expressed proteins in the mycelium and fruiting bodies. The color of the dots represents the adjusted *p*-value, and the size of the dots represents the number of differently expressed proteins that were identified in the pathway.

The KEGG pathway analysis classified the functions of proteins into 12 groups involved in different metabolic pathways of the mycelium, mainly including genetic information processing (18%), amino acid metabolism (15%), and carbohydrate and energy metabolism (13%). The proteins extracted from fruiting bodies were annotated into 10 groups, and the genetic information processing was significantly changed in the freezing-treated fruiting bodies (Figure 6A). We also analyzed the scatter plot of the significant KEGG enrichment function, and 20 and 15 pathways were enriched in the mycelium and fruiting bodies, respectively (Figure 6B). Among them, tyrosine metabolism, glycolysis/gluconeogenesis, ribosome, pantothenate and CoA biosynthesis, porphyrin and chlorophyll metabolism, cysteine and methionine metabolism, and oxidative phosphorylation showed co-enrichment in the mycelium and fruiting bodies. Glycolysis/gluconeogenesis, tyrosine metabolism, and arginine biosynthesis were mainly enriched in the mycelium. Ribosome, glycolysis/gluconeogenesis, and mRNA surveillance pathways were mainly enriched in the fruiting bodies. The key enzyme involved in the synthesis of all types of melanin from the initial precursor tyrosine is tyrosinase (Hearing, 2011), and tyrosine metabolism was mainly enriched in the mycelium. We infer that the melanin synthesis begins in the mycelium stage. This speculation was consistent with the enzyme activity results.

### UV–Visible/FTIR Spectra of *A. auricula-judae* Melanin Under Freezing Treatment

We conducted FTIR and UV analyses of melanin in the fruiting bodies after freezing treatment (Supplementary Figure 3). In the FTIR spectrum detection, we found that the wide peak of 400 cm^–1^ was the O–H stretching vibration of phenol. The absorption peak of 2950–850 cm^–1^ is the C–H stretching vibration of the aliphatic group, and the absorption peak of 1450 cm^–1^ is the C–C stretching vibration of the aliphatic group, indicating that there may be a small amount of protein in the two melanin species. The absorption peaks of 1600–1650 cm^–1^ were aryl skeleton C = C and carboxyl stretching vibration, and the peaks of 1380–1400 cm^–1^ were phenolic hydroxyl bending vibration and N–H stretching vibration in indole. In addition, the weak absorption peak within the range of 800–600 cm^–1^ indicates that aromatic rings are replaced, forming a conjugated system, and the content of aromatic hydrogen is low. However, the absorption peak of melanin in control and freezing treatments is not significantly different, possibly because it contains a small amount of proteins to cover up the differences in structure (Supplementary Figure 3A). The UV absorption spectra of the *A. auricula-judae* melanin are similar (Supplementary Figure 3B), showing a stronger absorption at 200–300 nm. In addition, the melanin has more absorption than the melanin under freezing treatment in the UV region, possibly because the former contains more eumelanin.

### EPR Analysis of *A. auricula-judae* Melanin Analysis

Electron paramagnetic resonance spectra were recorded using the X-band Bruker Elexsys 500 spectrometer (Karlsruhe, Germany) with 100-kHz field modulation and 9.849554-GHz microwave frequency at 77 K and at room temperature. The calculated *g* (2.004) indicates the presence of free radicals in the control and freezing treatment samples, which are the characteristic signals of all melanin and do not appear in any other biological tissues or non-melanin compounds that do not contain melanin (Supplementary Figure 4).

## Discussion

Freezing treatment is the most important step to cultivate *A. auricula-judae*, which is correlated with the growth rate, color formation, and biomass yield. Freezing treatment could improve the growth but negatively affect the color quality of fruiting bodies of *A. auricula-judae*. Until now, limited information has been available regarding the physiological and molecular mechanisms of how the freezing treatment impacts the growth and color of the mushroom. In our study, proteomic analysis was carried out, and the protein expression profiles of *A. auricula-judae* mycelium and fruiting bodies after freezing treatment showed that multiple biological processes, such as carbon metabolism, energy metabolism, biosynthesis of amino acids, protein processing and transport, and nucleotide metabolism, could be involved in the growth and color formation of *A. auricula-judae*.

**FIGURE 7:**
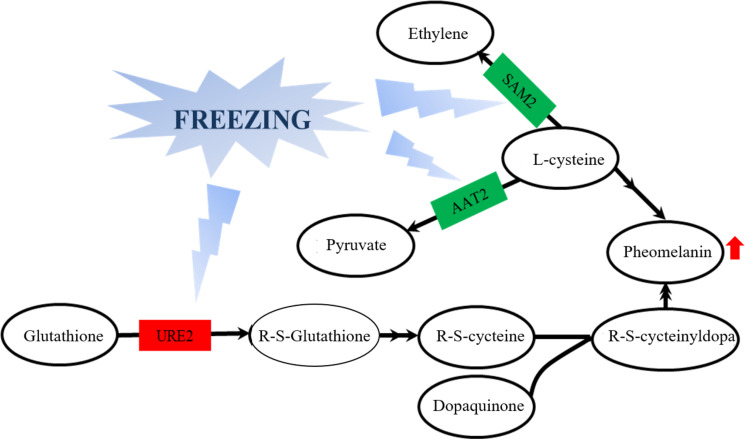
Effects of glutathione *S*-transferase, aspartate aminotransferase, and methionine adenosyltransferase on melanin metabolism of the *A. auricula-judae* mycelium under freezing treatment. The double arrow means the direct action and the single arrow means the indirect action happened between two factors in the nodes. Red represents upregulation, while green represents downregulation. AAT2, aspartate aminotransferase; URE2, glutathione *S*-transferase; SAM2, methionine adenosyltransferase.

From the observation of the color phenotype, the fruiting bodies obviously turn yellow after outdoor freezing treatment for 2 months (Figure 1A). In low-temperature environments, plants and fungi increase the activity of POD and SOD in the body, forming an active oxygen scavenging system to deal with the oxidative stress caused by low temperature (Zhang et al., 2013). The antioxidant enzyme involved in the regulation process of oxidative stress can oxidize the precursor, 2-*S*-cysteine DOPA quinone, and 5-*S*-cysteine DQ of pheomelanin, subsequently affecting the DOPA–melanin synthesis process (Galván and Alonsoalvarez, 2009). In the mycelium, the expression of manganese SOD (spot 149) was upregulated, which is consistent with the increase of the enzyme activity (Figure 2A). These results indicated that freezing treatment produces oxidative stress on the mycelium of *A. auricula-judae*. This may have some effects on the melanin synthetic process in the mycelium (Figure 2). The mixing ratio of melanin to eumelanin or pheomelanin during synthesis depends on the tyrosinase activity and substrate concentration of cysteine, which is involved in glutathione synthesis (Simon et al., 2009). Glutathione is implicated in the biogenesis of the pheomelanin (Benathan et al., 1999), and glutathione *S*-transferase (spot 154) is a key enzyme in the glutathione binding reaction that catalyzes the initial step of the reaction of glutathione binding (Chen et al., 2013), which may lead to the incremental accumulation of cysteine in the mycelium (Figure 7). When cysteine is present in the cell, DQ will immediately bind to cysteine to form cysteamine DOPA and then through multiple enzymatic reactions produce pheomelanin (Ito and Wakamatsu, 2003). As KEGG pathways show, methionine adenosyltransferase (SAM2) and aspartate aminotransferase (AAT2) are involved in cysteine metabolism and catalyze the reaction of aminoacrylate production, which is the precursor of L-cysteine. Our study found that the expression of SAM2 and AAT2 was downregulated after freezing treatment, and for support, the content of cysteine significantly decreased in the fruiting body (Supplementary Figure 5), which may influence the production of pheomelanin and the color quality of *A. auricula-judae* under freezing conditions (Figure 7). In addition, the increase of S element content after freezing treatment is consistent with the description of Zou et al. (2013), which indirectly proves that the synthesis of melanin after freezing treatment tends to synthesize pheomelanin, somehow indicating that the accumulation of pheomelanin may be induced by freezing and then brings about the color change of fruiting bodies.

Our study showed that differentially expressed proteins (spots 210, 214, 26, 242, and 341) in the mycelium were involved in the glycolysis/gluconeogenesis process (Table 1), which is the main process contributing to the high-efficiency production of biomass. The galactose mutarotation enzyme (spot 341) presented an 11.12-fold increase with the freezing treatment. Galactose induces cellulase production in lactose metabolism (Seiboth et al., 2002), which indicates that the utilization rate of the cultivated substrate in the mycelium was enhanced after freezing treatment. Methionine adenosyltransferase (spot 914) was identified as the GroES-like protein SFA1, which is involved in the glycolysis/gluconeogenesis pathway, and the increased abundance of SFA1 indicated that the utilization rate of fruiting bodies to the cultivated substrate was enhanced after freezing treatment (Yu et al., 2015). Among the differentially expressed proteins in the fruiting bodies (Table 2), phosphomannomutase (spot 285) was involved in the metabolism of fructose and mannose simultaneously and at the same time also involved in the protein posttranslational modification, providing the glycosylation proteoglycan core, which is the main component of the cell wall of the edible fungus (Seifert, 2004). Therefore, the upregulated expression of phosphomannomutase suggested that the cell wall of the fruiting bodies may be strengthened after the freezing treatment, resulting in a larger form of the fruiting bodies. Ribosomal protein L23 (spot 945), as a negative regulator in cell apoptosis (Wu et al., 2012), exhibited reduced expression after freezing treatment, which indicated that most of the cells in the fruiting bodies are mature. The expression of serine/threonine protein phosphatase (spot 36) in the fruiting bodies is upregulated after freezing treatment, which is an indispensable regulatory factor for the G2-M phase of the cell cycle in winemaking yeast (Thakur and Sanyal, 2011). The increased expression of serine/threonine-protein phosphatase may improve the mitotic activity in the fruiting bodies and then promote the growth and maturation of fruiting bodies. Ras-like protein (spot 90) is mainly located in the inner side of the cell membrane and plays an important role in the regulation of cell proliferation and cell metabolism (Yang, 2010). The greater expressed abundance of Ras-like protein indicated that the cell wall of fruiting bodies may be enlarged, leading to the larger morphology of fruiting bodies.

Branched-chain amino acids (BCAAs) are essential amino acids of the human body, and they are limited to plants and some microorganisms. They must be obtained through food and are valued for their nutritional applications (Shi and Yuan, 2003). BCAAs mainly include leucine, isoleucine, and valine. Ketol-acid reductoisomerases (spot 218) are a family of NADPH-dependent oxidoreductases, which are essential intermediates in the biosynthesis of BCAAs (Polaina, 1984). 3-Isopropylmalate dehydrogenase (spot 231) is a key enzyme for leucine biosynthesis, which is ultimately broken down into acetyl-CoA and acetoacetic acid and then enters the tricarboxylic acid cycle (Shmomura et al., 2006). Aspartic acid transaminase can convert phenylpyruvate (PPA) into L-phenylalanine in the presence of coenzyme pyridoxal phosphate (PLP) by using L-aspartic acid as the amino donor and PPA as the substrate. L-Phenylalanine is a nutritional supplement and one of the essential amino acids (Wei et al., 2000). The expression levels of ketol-acid reductoisomerases, 3-isopropylmalate dehydrogenase, and aspartic acid transaminase were significantly increased in the mycelium after freezing treatment, which indicated that the nutritional value of *A. auricula-judae* fungus might be increased after freezing treatment.

Overall, the present study provides evidence that tyrosinase and laccase impact the synthesis of melanin and then affect the color quality of *A. auricula-judae* fruiting bodies. The complete protein expression profile uncovered the underlying mechanisms of the modulation of the color quality and fruit growth of *A. auricula-judae* under freezing conditions. The proteins involved in glycolysis/gluconeogenesis, tyrosine metabolism, and ribosome and arginine biosynthesis likely play a key role in the synthesis of melanin, and the expression changes of several metabolic enzymes in these pathways might influence the accumulation of pheomelanin. These proteins can be used as molecular markers in gene engineering to improve the melanin synthesis of *A. auricula-judae* in extreme environments. This study has provided a valuable basis for further understanding the molecular mechanism of the effect of the color of *A. auricula-judae* after freezing treatment and fruiting bodies formation, and it also gives theoretical evidence for the quality control and industrial application of *A. auricula-judae*.

## Data Availability Statement

The datasets presented in this study can be found in online repositories. The names of the repository/repositories and accession number(s) can be found in the article/Supplementary Material.

## Author Contributions

QP, XY, and ZL conceived and designed the research. QP, XY, ZL, JL, and TZ conducted the experiments. ZL contributed the new reagents or analytical tools. ZL, JL, and TZ analyzed the data. JL wrote the manuscript. All the authors read and approved the manuscript.

## Conflict of Interest

The authors declare that the research was conducted in the absence of any commercial or financial relationships that could be construed as a potential conflict of interest.

## Footnotes

1 http://www.genome.jp/kegg/
