# Supplementary material for: Proteomic Analysis of Auricularia auricula-judae Under Freezing Treatment Revealed Proteins and Pathways Associated With Melanin Reduction
Source: Front Microbiol. 2021 Jan 15;11:610173. doi: 10.3389/fmicb.2020.610173 (PMC7844145; doi:10.3389/fmicb.2020.610173)
Supplement: Supplementary file 1 [file Data_Sheet_1.pdf]

**Table S1** Primer Sequences used in RT-PCR analysis.

| Gene name | Forward primer sequence (5'-3') | Reverse primer sequence (5'-3') |
|---|---|---|
| 18S | GCCGCTCCCTTGGTGATTCATAATA | CTTGGATGTGGTAGCCGTTTCT |
| hypothetical protein DICSQDRAFT_180523 | TAAAGAAAGGCGGTGAAGGA | AGGAGGTCGTATGGGAGGAT |
| hypothetical protein AURDEDRAFT_158505 | GCATCCCTGTTGTTTCGTCT | GGGCTTCTTCTTCCTCTGGTTCT |
| aspartate aminotransferase | CATAGAAGCGGCTGAGGAAG | CTGCTGGAGCTCGATAATCC |
| glutathione S-transferase | CGCATCTTCAACACATCGTT | GGAACTGCTTTCCTTTCACG |
| Transaldolase | AATTTCCTTGCCGAACTCAA | ACCAACCCGTCGCTTATTCT |
| Glutamate-cysteine ligase | ACGACTGGCCTTGATGTACC | GTCGAGGCTGCTTCTATTGG |
| Glutathione synthetase | TTCGCAAGACATACGACGAC | ATCTTCTTTGAGCCCGACAA |
| Tyrosinase | CCAGTACTGCGACTCGACCT | TGGAGAAATAGACCCGATGC |
| Laccase | GGCCAACACATTCGCTTATT | CCCGGGTTATCAGTCTTGAA |

**Table S2** Peptide sequences and observed m/z of the identified proteins response to acute stress in MALDI-TOF/TOF mass spectrometry analysis.

| Spot No. | Category and name | Peptide sequence | Observed m/z |
|---|---|---|---|
| 26 | iron-containing alcohol dehydrogenase 1, partial | K.EYAFEVAAANLR.F | 1353.681 |
| 218 | ketol-acid reductoisomerase | K.EVYSDLYGER.G | 1230.5645 |
| | | R.ELAEIDNQEIWR.A | 1515.7494 |
| 231/236 | 3-isopropylmalate dehydrogenase | K.VLDAPNDGGFGFR.T | 1364.693 |
| | | K.IAVLPGDGIGPEVVGEAVR.V | 1848.0747 |
| 241 | aspartate aminotransferase | K.YHIYLTSNGR.I + Deamidated (NQ) | 1224.5925 |
| | | K.NVGITPVDYAYYDPR.T | 1742.8257 |
| 242 | NAD(P)-binding protein | K.AIQISEHGGPEVLR.L | 1505.7863 |
| | | K.VDYAGVNFIDNYQR.S | 1673.7609 |
| 268 | acetylglutamate kinase ARG6 | R.EGVVPDYIDGIR.I | 1332.6854 |
| | | R.SLFWYGVQDVDEVER.I | 1841.8995 |
| 328 | trehalose phosphorylase | K.RPELPIIYR.S | 1156.6814 |
| | | K.NYAHDICVMR.L | 1278.5746 |
| | | K.SGFTSLPSQEIR.R | 1321.6752 |
| | | K.CVMFFGPNNNPR.L | 1452.6542 |
| | | R.HLYQLWTDDALYDR.M | 1808.8655 |
| 27 | citrate synthase | R.ASLPEFVEEIIDR.C | 1517.777 |
| | | K.TFSPVQVDQLYGGMR.G | 1697.8284 |
| 33 | transaldolase | K.IASTWEGIQAAR.V | 1302.6738 |
| 210 | fructose-1,6-bisphosphatase | R.TLLYGGIFGYPDDR.K | 1586.776 |
| 214 | Thiamin diphosphate-binding protein | R.SIRPLDIDTIK.A | 1270.7117 |
| | | R.DETVYILGEEVAR.Y | 1493.725 |
| | | R.EALNSAMEEEMLR.D | 1522.6676 |
| | | K.TYYMSGGNVPCPVVFR.G | 1846.8385 |
| 284 | hypothetical protein AURDEDRAFT_90114 | K.LGHPDGELNLTR.A | 1321.7205 |
| | | R.ALEILHGELEMNLR.L | 1637.8945 |
| | | K.AWAYYSSAADDEITHR.E | 1855.866 |
| | | K.LVSWYDNEWGYSAR.V | 1745.7975 |
| 74/81 | V-type ATPase | K.RTTLVANTSNMPVAAR.E + Deamidated (NQ) | 1718.8457 |
| 91 | V-type ATPase | K.RTTLVANTSNMPVAAR.E + Deamidated (NQ) | 1718.7948 |
| 385 | pyrophosphatase-domain-containing protein | K.YATEIVHECHEAWR.R | 1800.8318 |
| 159 | ribosomal protein L13e | K.TWFNQPGR.K | 1005.4773 |
| | | R.HNNVLPANHFR.K | 1318.6576 |
| | | R.AALPLPAPSAPEAPR.K | 1457.827 |
| 195 | ubiquitin, partial | K.EGIPPDQQR.L | 1039.5022 |
| | | K.IQDKEGIPPDQQR.L | 1523.7734 |
| | | K.TITLEVESSDTIDNVK.A | 1763.8671 |
| 211 | Bet v1-like protein | R.DFVGVQLVER.G | 1161.6179 |
| | | R.TVATEIPMCTGR.V | 1335.6337 |
| 217/280 | "eukaryotic translation initiation factor 2 subunit alpha" | K.YPEVDELVMVQVR.Q | 1576.7987 |
| 247 | 26S proteasome subunit P45 | R.EVIELPLLNPELFQR.V | 1810.0293 |
| 306 | hypothetical protein AURDEDRAFT_178815 | K.AHLYAFYER.T | 1169.5787 |
| 341 | galactose mutarotase-like protein | K.GYDSFYVFR.D | 1153.5187 |
| | | K.EIALGSLVILYR.A | 1346.7836 |
| 144 | hypothetical protein AURDEDRAFT_158505 | R.AMTPTQWESLR.F + Deamidated (NQ) | 1320.7026 |

| Spot No. | Category and name | Peptide sequence | Observed m/z |
|---|---|---|---|
| | | R.AMTPTQWESLR.F + Deamidated (NQ) | 1336.7227 |
| | | R.VSLIEVIPDNIR.Y | 1367.9075 |
| 206 | NAD(P)-binding protein | K.SITESLAHELR.A | 1255.6553 |
| 149/365 | manganese superoxide dismutase | K.NVRPDYLK.A | 1004.5404 |
| | | K.HHQAYINALK.T | 1194.6353 |
| | | K.FNGGGHINHSLFWK.N + Deamidated (NQ) | 1614.776 |
| 83 | glycoside hydrolase family 38 protein | R.FDAHVDWHQR.H | 1310.601 |
| 49 | prolyl aminopeptidase serine peptidase | R.IENHYFINEGFMR.Q | 1669.7778 |
| 320 | hypothetical protein AURDEDRAFT_153044 | K.EFFFMEMNTR.L | 1351.6025 |
| | | R.TVAVYSDADANALHVR.L | 1701.8766 |
| 154 | glutathione S-transferase | K.LKDVPFDTIK.S | 1175.6345 |
| 182 | Isopentenyldiphosphate isomerase | R.AFSAFVFRPSDGK.L | 1428.7318 |
| | | R.DFLFGWWDELLK.R | 1568.7753 |
| 233 | GTP binding protein | R.EIDIELMR.R | 1018.5397 |
| | | R.EYDHVLAEESR.I | 1347.6447 |
| | | K.SLTHDVLYETYR.T | 1496.7627 |
| | | R.IYPWGVVEVDNPK.H | 1515.8024 |
| | | R.IKPVNIELEEDGVR.I | 1610.9055 |
| 85 | YjgF-like protein | K.FFEGVVPPAR.T | 1118.5895 |
| 88 | hypothetical protein AURDEDRAFT_63592 | K.SWLQESAVWAVLR.A + Dioxidation (W) | 1576.6932 |
| | | R.KSWLQESAVWAVLR.A + Dioxidation (W) | 1704.7516 |
| 99 | cyclophilin | K.HVVFGEVVEGK.N | 1199.6313 |
| | | K.LTHDRPYLLSMANAGK.N | 1786.927 |
| | | R.ELATGQHGFGYAGSSFHR.V | 1921.925 |
| 208 | hypothetical protein AURDEDRAFT_126065 | R.LLACNILVSR.H + Deamidated (NQ) | 1159.569 |
| 223 | protein prenylyltransferase | K.NYQVWHHR.R | 1139.5551 |
| | | R.LNPAHYTVWQYR.W | 1547.7816 |
| | | K.IALAPNNESAWNYLR.G | 1731.8876 |
| 228 | putative cyanide hydratase | K.VTYQQSLPMLK.S | 1307.6884 |
| | | R.ENSLPVDSEEMR.R | 1405.6337 |
| | | K.VLADFGGHYTRPDLIR.L | 1829.9751 |
| 239 | carbohydrate-binding module family 12 protein | R.YNIIQPHR.S | 1040.553 |
| | | R.AYHEGGIQVGK.A + Deamidated (NQ) | 1159.5779 |
| | | K.NWFDLSDER.K + Deamidated (NQ) | 1182.501 |
| 243 | Nuclear and cytoplasmic polyadenylated RNA-binding protein | R.SAFQDFGQILDSIVMR.D | 1826.9351 |
| 255 | proliferation-associated protein 1 | K.AGPFPFTLR.C | 1005.5376 |
| | | K.NFAVTDAVNR.A | 1106.5427 |
| | | R.IVLNPTEQQR.R | 1197.6433 |
| 36 | serine/threonine-protein phosphatase PP1 | K.YPENFFILR.G | 1198.601 |
| | | K.ICGDIHGQYYDLLR.L | 1722.7968 |
| | | R.GNRPGKPVQLQEYEIK.Y | 1855.9692 |
| 99 | eukaryotic translation initiation factor 2 subunit alpha | R.AGEAVSTDAVPIR.A | 1285.6993 |
| 667 | L30e-like protein | K.NALVHDGLAR.G | 1065.5758 |
| | | K.LIEALCAEHK.I | 1183.6068 |
| | | K.LSVEDALQQVLK.N | 1342.7501 |
| 732 | guanine nucleotide binding protein beta subunit | K.TIIVWELTR.G | 1130.6656 |
| | | R.LWDLNTGLTTR.T | 1289.6998 |
| | | K.QVVAELVPETTEATR.V | 1642.8893 |

| Spot No. | Category and name | Peptide sequence | Observed m/z |
|---|---|---|---|
| 742 | serine/threonine specific protein phosphatase Sit4 | K.LSYSFLQFDPAPR.A | 1540.7654 |
| | | R.QITQVYGFYDECLR.K | 1791.8245 |
| | | R.AHQLVMEGYNWSQDR.N | 1833.8215 |
| 865 | 40S ribosomal proteinS3 | R.AELNEFFTR.E | 1126.5336 |
| | | R.GLSAVAQCESLR.Y | 1290.6256 |
| | | R.ELAEEGYSGCDVR.V | 1484.6124 |
| | | K.FTDGFMIHSGQPSR.D | 1579.7036 |
| | | K.FTDGFMIHSGQPSR.D + Oxidation (M) | 1595.6984 |
| | | R.FKFPENSLELYAEK.V | 1714.828 |
| 872 | hypothetical protein AURDEDRAFT_167263 | K.EPILQENHPFLR.Q | 1492.7831 |
| 909 | 60S ribosomal protein L35 | K.QLEELKGELLELR.V | 1569.8835 |
| 945 | ribosomal protein L14b/L23e | K.ECADLWPR.I | 1046.5142 |
| | | K.NLYIIETFGFGAR.L | 1500.8135 |
| 90 | ras-like protein | R.INVDEAFSSLVR.E | 1349.7128 |
| | | R.NSFEEISTFHQQILR.V | 1848.9222 |
| 285 | phosphomannomutase | R.NGMINVSPIGR.N + Deamidated (NQ) | 1158.574 |
| | | K.EAFDEIHFFGDR.T | 1482.6428 |
| 430 | GroES-like protein | R.WCQLEGAR.R | 1019.4474 |
| | | R.VVAIDNVPER.L | 1111.5837 |
| | | R.GLSSMCDTTNNTAIAGR.M | 1768.7694 |
| | | R.LLEMESYGWDCCIDAAAFR.Y | 2306.9675 |
| 754 | glyceraldehyde 3-phosphate dehydrogenase | R.VPVADVSVVDLVVR.L | 1466.849 |
| | | K.LVIDGHPIAVFAER.D | 1536.8525 |
| | | K.LVSWYDNEWGYSAR.V | 1745.7975 |
| 914 | methionine adenosyltransferase | R.WCQLEGAR.R | 1019.469 |
| | | R.VVAIDNVPER.L | 1111.6045 |
| | | R.LLEMESYGWDCCIDAAAFR.Y | 2306.9866 |
| 309 | hypothetical protein DICSQDRAFT_180523 | R.RAEEDAQAALEAIR.A + Deamidated (NQ) | 1543.8517 |
| 962 | Ketol-acid reductoisomerase | K.EVYSDLYGER.G | 1230.5645 |
| | | R.ELAEIDNQEIWR.A | 1515.7494 |
| 942 | V-type ATPase | K.RTTLVANTSNMPVAAR.E + Deamidated (NQ) | 1718.8098 |
| 955/957 | hypothetical protein AURDEDRAFT_158505 | R.AMTPTQWESLR.F | 1319.6320 |
| | | R.AMTPTQWESLR.F + Oxidation (M) | 1335.6228 |
| 958 | cleavage and polyadenylation specific factor 5 | K.LLAIPLFELYDNAAR.Y | 1718.9292 |
| 961 | NAD(P)-binding protein | K.WEVTIYGR.E | 1023.5314 |
| 75 | peptidase C14 | K.QEDIVMLLDGLANPR.Q | 1683.8553 |
| 141 | chaperonin Cpn10 | K.IGEDEYHIFK.D | 1250.582 |
| 665/944 | cyclophilin | R.ELATGQHGFGYAGSSFHR.V | 1921.8885 |
| 852 | E set domain-containing protein | K.TVEEYAALDAEDESLAR.W | 1881.8735 |
| 864 | voltage-dependent ion-selective channel | K.GPTFTADTVLGR.D | 1234.641 |
| | | R.DGFLVGLEGSYNVSEGR.I | 1798.8610 |
| 919 | hypothetical protein AURDEDRAFT_110532 | K.VAADFAEGEPTSETLAIR.F | 1876.9283 |
| 960 | hypothetical protein AURDEDRAFT_92020 | R.CVTTYAGVSHTQLAR.D | 1663.8335 |
| | | K.YVLDDWEGAPDSFVCQEQR.Q | 2314.0122 |
| 26 | iron-containing alcohol dehydrogenase 1, partial | K.EYAFEVAAANLR.F | 1353.681 |
| 218 | ketol-acid reductoisomerase | K.EVYSDLYGER.G | 1230.5645 |

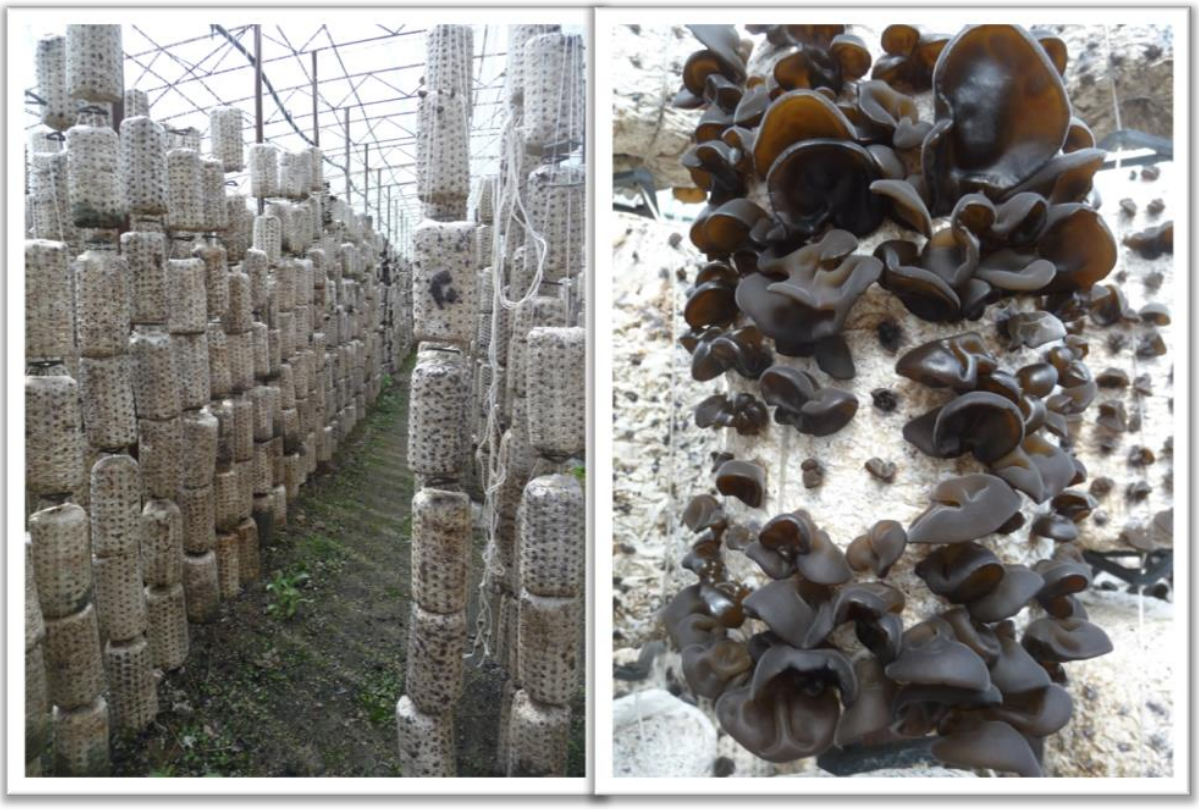

**Supplementary Figure 1.** Bagging cultivation of *A. auricular-judae*. The mycelium is attached to a bag filled with sterile sawdust (Left). Under the proper cultur conditions, the fruit body of *A. auricular-judae* will grow out from the holes on the bag (Right).

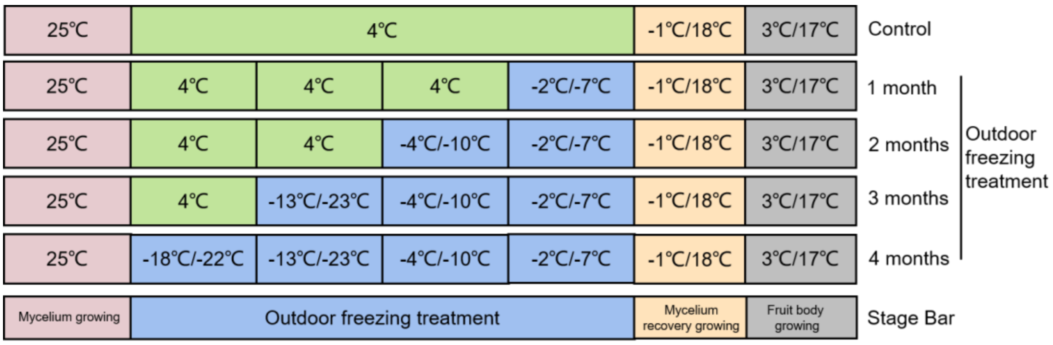


**Supplementary Figure 2.** The design of the freezing treatment into *A. auricular-judae*. The mycelium grow (pink column) at 25°C for 2 months, then stored at 4°C (green column) for one, two, three and four-month following by move to outdoor as three, two, one month-freezing treatment (blue column) respectively, or directly move to outside for 4-month freezing treatment, then *A. auricular-judae* goes to mycelium recovery growing (yellow column) stage. After 15 days of that, the fruiting bodies grow (gray column) in natural temperature between 3°C and 17°C for 80 days.

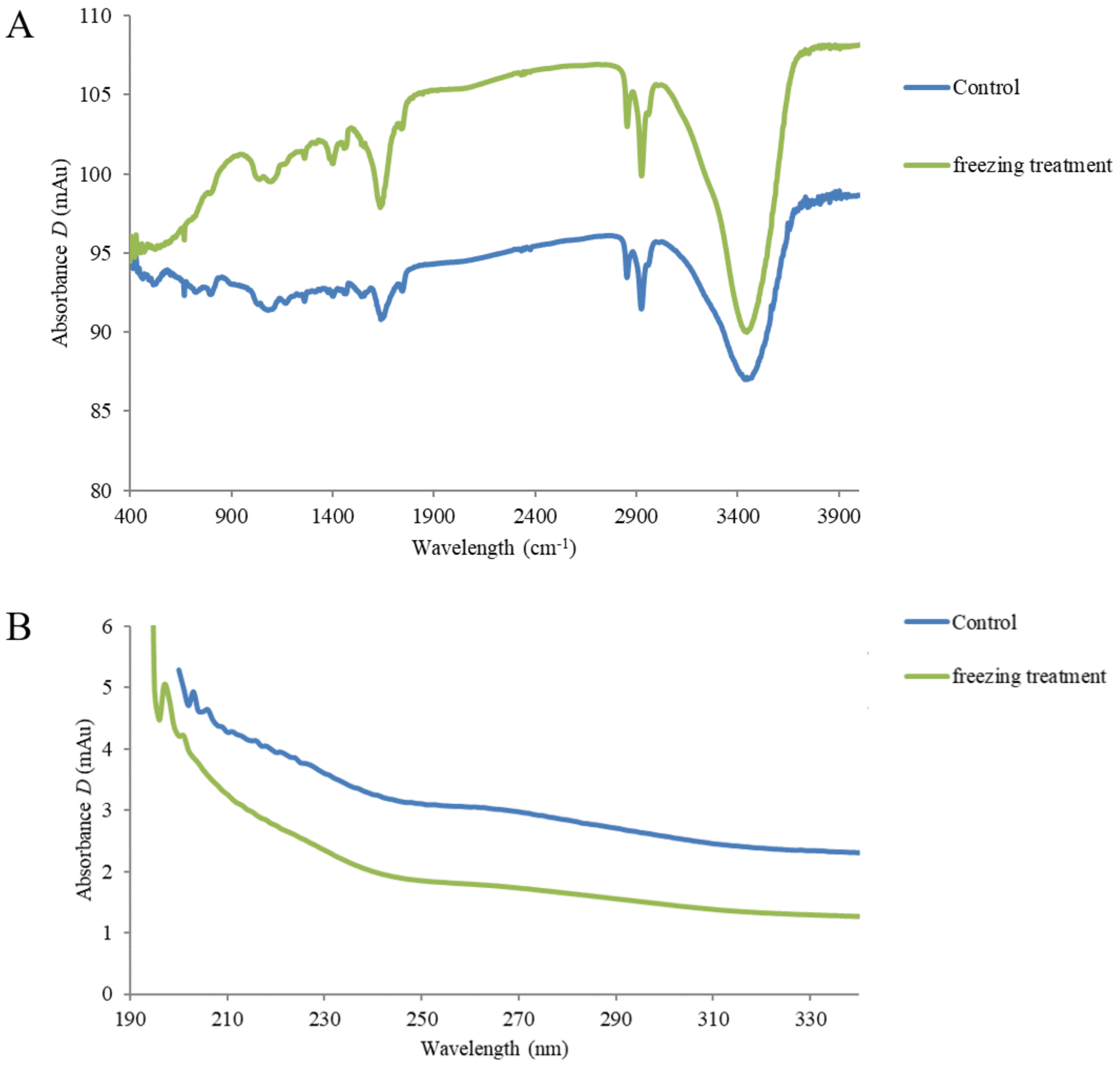


**Supplementary Figure 3.** FTIR/UV-visible spectra of *A. auricula-judae* melanin in fruiting bodies under freezing treatment.

**(A)** IR spectra of *A. auricula-judae* melanin in fruiting bodies under freezing treatment. **(B)** UV-visible spectra of *A. auricula-judae* melanin in fruiting bodies under freezing treatment.

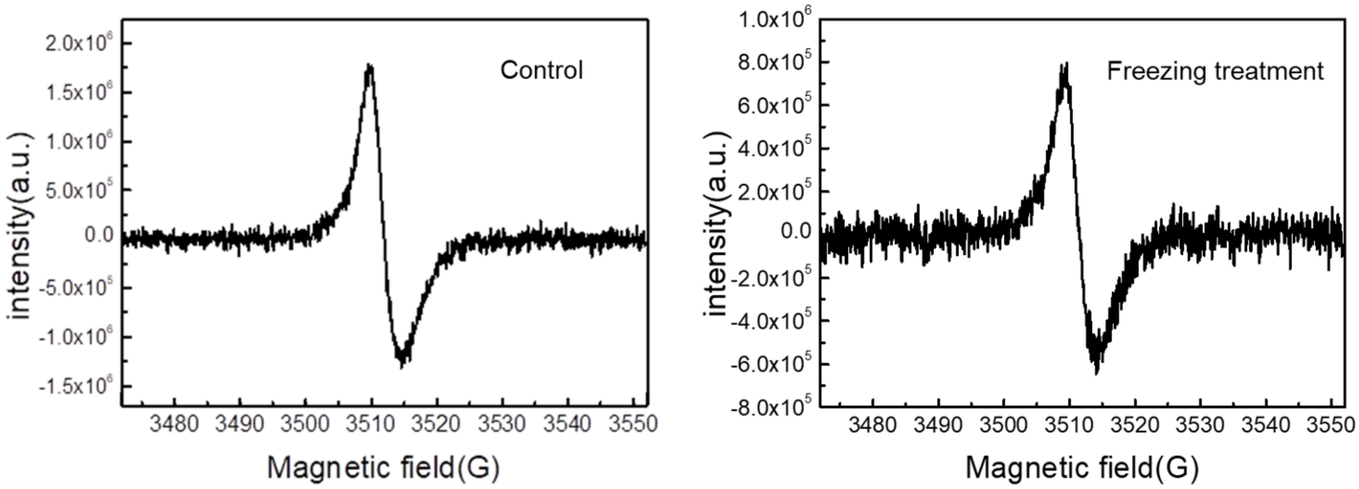


**Supplementary Figure 4.** EPR spectra of *A. auricula-judae* melanin in fruiting bodies under freezing treatment.

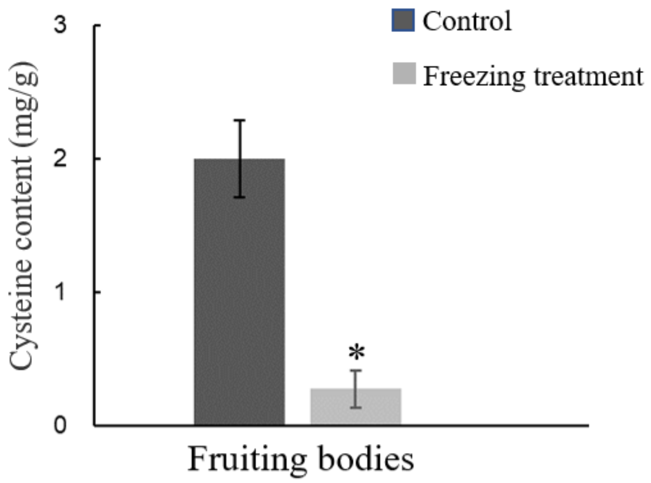


**Supplementary Figure 5.** The content of cysteine and melanin after freezing treatment in *A. auricular-judae*. * represent a significant level of difference ($p \leq 0.05$).
